# The impact of learning engagement on learning outcomes among public administration majors: the mediating role of digital literacy and the moderating role of AI tool acceptance

**DOI:** 10.3389/fpsyg.2026.1857564

**Published:** 2026-06-10

**Authors:** Jihui Liu, Yan Qiao, Jianda Zhou, Chuhan Fu, Tongxin Wang, Xinnan Li, Renhe Wang

**Affiliations:** 1 College of Humanities and Social Sciences, Hebei Agricultural University, Baoding, China; 2 School of Ethnology and Sociology, Minzu University of China, Beijing, China

**Keywords:** AI tool acceptance, digital literacy, learning engagement, learning outcomes, UTAUT

## Abstract

To explore the relationship between learning engagement and learning outcomes among students majoring in public administration, and to analyze the mediating role of digital literacy in both, as well as the moderating effect of AI tool acceptance on learning engagement and digital literacy. This study employed the learning engagement scale, digital literacy scale, AI tool acceptance scale, and learning outcomes scale, surveying 400 students majoring in public administration. Data analysis was conducted using the PROCESS model in SPSS software and Mplus. The study found that learning engagement positively influenced learning outcomes (*β* = 0.342, *p* < 0.01); learning engagement positively influenced digital literacy (*β* = 0.418, *p* < 0.01); digital literacy positively influenced learning outcomes (*β* = 0.374, *p* < 0.01); digital literacy played a mediating role in the relationship between learning engagement and learning outcomes (*β* = 0.235, *p* < 0.01); AI tool acceptance positively regulated the relationship between learning engagement and digital literacy (*β* = 0.204, *p* < 0.01). These findings suggest that in the teaching of public administration majors, it is necessary to emphasize the cultivation of students’ digital literacy and encourage their positive acceptance of AI tools to enhance learning outcomes.

## 1 Introduction

With the global spread of intelligent technology led by generative artificial intelligence (GenAI), various sectors of society have undergone tremendous changes due to its integration, and the education sector is experiencing a profound transformation. Artificial intelligence is not merely an instrument but a transformative force, reshaping the ways knowledge is produced, transmitted, and acquired (Luckin et al., 2016). From the automatic generation of personalized learning paths to the widespread application of intelligent tutoring systems, the role of AI in education has become increasingly prominen (Zawacki-Richter et al., 2019). For instance, studies have shown that collaboration between AI systems and human teachers can significantly enhance learning outcomes, which is driving the adjustment and innovation of traditional teaching models (Holstein et al., 2018). As a frontier field studying public sector governance, policy processes, and public services, public administration has also been impacted by digitalization and intelligent technologies in terms of its knowledge system and practical skill requirements. Policy simulation, intelligent analysis of public opinion, and big data–driven performance evaluation have become core scenarios of modern public governance (Mergel et al., 2019). This implies that future public administrators not only need to master traditional administrative knowledge but also must possess digital literacy to cope with a digitally oriented working environment (Ravšelj and Aristovnik, 2025) and be able to uphold ethical communication and institutional responsibility awareness in AI applications (Miluniec et al., 2025). Therefore, cultivating new types of management talents who possess digital literacy and can use AI tools to address complex, contemporary public problems has become an urgent task in educational reform. This study is grounded in this practical need, focusing on public administration students as the main subjects, and aims to explore the mechanisms through which learning engagement, digital literacy, and AI acceptance influence learning outcomes, thereby providing a scientific basis for teaching reform in this field.

In the field of education, researchers have conducted studies from multiple perspectives. Some studies, based on the integrated framework of the Technology Acceptance Model (TAM) and self-determination theory, have found that ChatGPT use behavior has a significant positive impact on learning motivation (Susanto et al., 2025; Eccles and Wigfield, 2002). The positive effect of students’ learning engagement on learning outcomes has been empirically established. Studies have shown that learning engagement promotes college students’ academic achievement and is a key factor influencing academic success (Carini et al., 2006). Another study indicates that factors such as teacher–student interaction, social media use, family support, and technical support positively influence students’ learning performance through their engagement (Kedia and Mishra, 2023). It can be inferred that AI technology may also affect learning outcomes via learning engagement. In online learning, learning engagement has a significant influence on learning outcomes and teaching quality (Yu, 2022). Additionally, some scholars have explored students’ abilities to effectively acquire, evaluate, and create information in digital environments from the perspective of digital literacy (Ng, 2012). Empirical research also shows that digital literacy has a significant positive effect on learning outcomes, and that the interaction between digital literacy and learning motivation also has a significant positive effect on learning outcomes (Puniatmaja et al., 2023). However, little research has examined how AI technology specifically empowers the learning process of students in particular fields—especially public administration—and how it enhances their learning outcomes. A large body of educational practice has shown that simply introducing AI tools does not necessarily lead to improved learning outcomes (Zawacki-Richter et al., 2019). Issues related to students’ technology acceptance and their learning engagement may serve as key mediating factors. In this logical chain of digital technology–empowered learning improvement, whether students’ digital literacy can moderate the effectiveness of this process is also a question that needs to be answered.

Although existing research has laid a foundation for understanding AI applications in education, significant limitations remain. In-depth empirical studies on how AI technology specifically empowers the learning process of students in specific fields (especially public administration) and brings about substantive improvements in learning outcomes are scarce. Students’ digital literacy and learning engagement may be key mediating factors, yet the existing literature does not provide sufficient analysis of the internal transmission mechanism of “learning engagement → digital literacy → learning outcomes.” Although studies have shown that the degree of learning engagement directly affects its utility in learning, the specific mechanisms in different disciplinary contexts still need to be explored in depth (Carini et al., 2006). In terms of digital literacy, some scholars have pointed out that in the era of artificial intelligence, digital literacy requires particular emphasis on ethical judgment and critical evaluation abilities (Hu, 2026), but the moderating role of these abilities in the pathway through which learning engagement affects learning outcomes remains empirically unexplored. Therefore, incorporating digital literacy as a mediating variable into the model holds important theoretical value and practical significance. To reveal the underlying relationships and logic, this study takes public administration majors as the research subjects. Based on the theoretical framework of the Unified Theory of Acceptance and Use of Technology (UTAUT2) and learning engagement theory, it introduces “learning engagement” as the core mediating variable and constructs a conceptual model to explain how AI enhances learning outcomes. This study aims to answer three core questions: (1) How does students’ learning engagement directly affect their learning outcomes? (2) What role does digital literacy play in this pathway? (3) Does AI tool acceptance moderate this influence process? Through empirical testing of these questions, this study hopes to provide empirical and theoretical insights into the internal mechanisms underlying the deep integration of AI and professional education, and to offer a scientific basis for decision-making in purposefully implementing AI-enabled teaching reform in public administration programs.

## 2 Theory and hypotheses

In the context of public administration, the introduction of AI tools is intended not only to enhance learning efficiency but also to influence students’ understanding of policy analysis, public value judgment, and governance logic. The teaching objectives of public administration programs emphasize the integration of theory and practice, with a particular focus on students’ adaptability and critical thinking in the realm of digital governance. Consequently, in an AI-enabled teaching environment, the nature and mechanisms of learning engagement may exhibit characteristics that differ from those observed in traditional educational settings. Drawing on established theories and accounting for the distinct features of the public administration discipline and the AI-mediated teaching context, this section proposes the following hypotheses.

### 2.1 The relationship between learning engagement and learning outcomes

In the AI-enabled teaching context of public administration programs, students’ learning engagement is reflected not only in their mastery of course content but also in their active participation in professional tasks—such as policy analysis, case simulation, and data interpretation—that are assisted by AI tools. Therefore, in this context, the effect of learning engagement on learning outcomes may be more complex and yet more critical. Learning engagement refers to a positive, fulfilling, and learning-related mental state (Schaufeli et al., 2002). Based on a critical review integrating the perspectives of critical reflection, psychology, sociocultural theory, and holism, a conceptual framework that synthesizes the strengths of each perspective has been proposed. This framework places students at the core and includes three psychological dimensions of engagement—emotion, cognition, and behavior—reflecting the psychological energy and level of behavioral participation individuals invest in the learning process (Kahu, 2013). Learning outcomes typically refer to the improvements and changes that learners achieve in knowledge, skills, attitudes, or values through participating in learning activities (Yin et al., 2024). A large body of research has demonstrated that learning engagement is a key factor influencing academic success and can help college students achieve good academic outcomes (Carini et al., 2006).

In the academic community, the positive impact of learning engagement on learning outcomes has been widely accepted as a consensus in educational research. Further studies have revealed that the association between academic achievement and behavioral engagement is the strongest, followed by cognitive engagement and then emotional engagement. This finding suggests that the contributions of different dimensions of learning engagement to learning outcomes vary, yet all show a significant positive effect overall (Wong et al., 2024). In higher education, some studies have concluded that learning engagement is a key predictor of college students’ academic achievement, and its importance becomes even more prominent with the widespread adoption of digital learning environments (Carini et al., 2006). Other research has found effect size differences in the relationship between learning engagement and learning outcomes, with graduate students showing the largest effect size, followed by undergraduate students and adult learners (Young and Jungwon, 2024). Studies focusing on specific types of learning engagement have revealed that behavioral, cognitive, emotional, and proactive engagement have differentiated predictive effects on academic achievement, social support, motivation, and wellbeing (Reeve et al., 2025), further confirming that each dimension of learning engagement positively influences various outcomes, including academic achievement. In digital higher education, some scholars have discovered and clearly pointed out that students’ level of engagement in digital environments is significantly correlated with their academic performance and emotional experiences (Li et al., 2026). From a longitudinal causal perspective, research has found that sustained high levels of engagement—whether occurring independently or in combination with high academic achievement—are reliable indicators of students maintaining a stable academic trajectory. Students who remain engaged typically achieve higher levels of academic success. More importantly, among students with low engagement, disengagement at any point is consistently associated with lower academic achievement, whereas students who increase to a higher level of engagement can at least achieve acceptable academic outcomes with a very low dropout rate (Saqr et al., 2023). This longitudinal study provides strong evidence for the causal relationship that learning engagement positively influences learning outcomes.

Based on the empirical findings summarized above, learning engagement has been proven to be an important factor positively influencing learning outcomes, regardless of whether the context is traditional higher education, online learning, or digital higher education environments. Students with higher levels of learning engagement are more likely to achieve better learning outcomes. Therefore, this study proposes the following hypothesis:

*H1:* In the context of AI embedded in teaching, learning engagement positively influences learning outcomes.

### 2.2 The mediating role of digital literacy

The integration of AI tools into the teaching of public administration–related courses requires a certain level of digital literacy. In this context, digital literacy primarily refers to the comprehensive capacity to acquire, assess, synthesize, and critically interpret policy-related information within a digital governance environment. Given that public administration students are likely to encounter governance scenarios involving algorithmic decision-making and digital government in their future careers, their level of digital literacy directly influences their ability to effectively apply AI tools to public administration and governance practice. Consequently, digital literacy may serve as a critical mediating variable between learning input and learning outcomes. Digital literacy refers to an individual’s ability to effectively acquire, process, create, and communicate information in a digital environment. It goes beyond mere technical operational skills and encompasses multi-dimensional capabilities such as critical thinking, problem-solving, and ethical judgment (Widowati et al., 2023). In the era of artificial intelligence, digital literacy requires particular emphasis on ethical judgment and critical assessment capabilities (Hu, 2026). In the context where digital technology is fully integrated into the education sector, digital literacy has become a key variable influencing students’ learning processes and learning outcomes.

A study employing the Unified Theory of Acceptance and Use of Technology 2 (UTAUT2) model found that students’ behavioral intention to use digital learning resources positively predicted their digital literacy level. This finding indicates that students’ actual usage behavior and underlying intentions in the learning process are important drivers for enhancing digital literacy (Liu et al., 2025). Learning engagement, as an internal driving force for actual learning behaviors (Xu et al., 2023), directly affects the depth of students’ use of digital learning resources (Baba and Misdi, 2025), and thereby influences the formation and improvement of digital literacy. From the above studies, it can be observed that learning engagement not only enhances learning outcomes but also promotes the formation and development of digital literacy. Students with higher levels of learning engagement are more likely to accumulate rich experience in using AI tools in practice, thereby improving their abilities to acquire, evaluate, and create information in digital environments.

The positive impact of digital literacy on students’ learning outcomes has been affirmed in both analytical and empirical research. Research on the relationship between digital literacy and university students’ academic performance indicates a moderately significant positive correlation, with satisfactory heterogeneity test results and no publication bias (Ardhiani et al., 2023). In the higher education context, studies have emphasized the importance of developing sufficient digital capabilities, which affect students’ learning and their post-class improvement (Cabero-Almenara et al., 2022). This provides further evidence that digital literacy enhances learning outcomes. In the field of online learning, studies have demonstrated that university students’ information literacy and online learning engagement are significantly correlated with online learning effectiveness, and the positive fit effect of information literacy on online learning engagement in turn influences online learning effectiveness (Su et al., 2023). This is an important study demonstrating the mediating role of digital literacy in influencing learning outcomes. Similarly, in blended learning contexts, digital literacy tools have been effectively used to measure and predict students’ learning outcomes (Lukitasari et al., 2022).

In research on digital learning abilities, scholars have found that digital learning ability has a significant positive indirect effect on academic achievement through informal digital learning engagement, providing indirect evidence for the mediating role of digital literacy between learning engagement and learning outcomes (Ko, 2026). Other studies have found that digital literacy significantly affects students’ engagement and perception of educational quality, further revealing the important bridging role of digital literacy between student engagement and educational quality (Phon and Ali, 2025). In the context of public administration education, the transmission effect of digital literacy is particularly prominent. Students majoring in public administration will enter government, the public sector, or social organizations in the future. Their work scenarios require practitioners to have a critical understanding of algorithmic decision-making and data-driven governance, rather than merely operational skills (Mergel et al., 2019). Therefore, with increased learning engagement, a higher level of digital literacy can be developed, which can more effectively integrate AI tools with public administration knowledge and skills, thereby achieving more significant improvements in learning outcomes.

Thus, this study proposes the following hypotheses:

*H2:* In the context of AI embedded in teaching, learning engagement positively affects digital literacy.

*H3:* In the context of AI embedded in teaching, digital literacy positively affects learning outcomes.

*H4:* In the context of AI embedded in teaching, digital literacy plays a mediating role between learning engagement and learning outcomes.

### 2.3 The moderating role of AI tool acceptance

In the teaching of public administration–related courses, students’ AI tool acceptance is shaped not only by perceived ease of use but also by deeper considerations regarding the legitimacy, accountability, and ethical boundaries of these technologies. Consequently, students in public administration tend to exhibit a more cautious stance toward adopting AI. Against this backdrop, whether AI acceptance can positively moderate the relationship between learning engagement and digital literacy emerges as a significant question. AI tool acceptance (Artificial Intelligence Tool Acceptance) in academic research typically refers to the positive attitudes that individuals or groups hold toward artificial intelligence technologies and their applications, as well as their inclination to incorporate them into their own usage intentions or actual usage behaviors (Koenig, 2025; Kelly et al., 2022). The Technology Acceptance Model (TAM) indicates that perceived usefulness and perceived ease of use are key factors driving actual usage behavior (Davis, 1989). Building on this foundation, TAM was theoretically extended by introducing social influence and cognitive instrumental processes (Venkatesh and Davis, 2000). Subsequently, scholars proposed the Unified Theory of Acceptance and Use of Technology (UTAUT). The UTAUT model, with its core concepts of performance expectancy, effort expectancy, social influence, and facilitating conditions, posits these as direct determinants of usage intention and behavior (Venkatesh et al., 2003).

Based on conservation of resources theory, research has found that the use of AI in teaching affects students’ creativity through the mediating role of learning engagement, and that AI literacy plays a moderating role between AI usage and learning engagement (Zhou and Peng, 2025). This indicates that individuals’ cognitive literacy and usage attitudes toward AI technology play an important moderating role in the learning engagement process. In international research, numerous studies have examined the moderating effect of digital literacy. For instance, some research has found that AI literacy and AI self-efficacy can explain the relationship between attitude and AI acceptance, confirming the moderating role of AI-related literacy in the technology acceptance process (Türk et al., 2025). Regarding the relationship between AI tool acceptance and learning engagement, it has been found that perceived usefulness and perceived ease of use—two components of AI tool acceptance—have a significant positive impact on learning engagement (Chen and Dong, 2026), indicating a close correlation between AI tool acceptance and learning engagement. In the relationship between AI tool acceptance and digital literacy, a study integrating the digital capability framework with UTAUT2 found that digital capability significantly affects students’ acceptance and use of ChatGPT (Caner-Yıldırım, 2025) suggesting an interactive relationship between AI tool acceptance and digital literacy. Other scholars have clearly pointed out that technology acceptance must be supported by digital literacy skills in order to achieve successful technology use in learning (Saregar et al., 2024). This finding indicates a close, mutually reinforcing relationship between technology acceptance and digital literacy. Moreover, in research on adaptive learning technology and student engagement, researchers treated digital literacy as a moderating variable, emphasizing its important role in influencing the relationship between antecedent variables and engagement (Yaseen et al., 2025). Although these studies did not directly test the moderating effect of AI tool acceptance on the relationship between learning engagement and digital literacy, they provide sufficient theoretical and empirical evidence to support the existence of such a moderating effect. Based on the above theoretical reasoning and indirect evidence, when students have a high level of AI tool acceptance, they will show a stronger positive effect in promoting the improvement of digital literacy as their learning engagement increases. In other words, the positive impact of learning engagement on digital literacy will strengthen as AI tool acceptance improves.

Accordingly, this study proposes:

*H5:* In the context of AI embedded in teaching, AI tool acceptance positively moderates the relationship between learning engagement and digital literacy.

**Figure 1:**
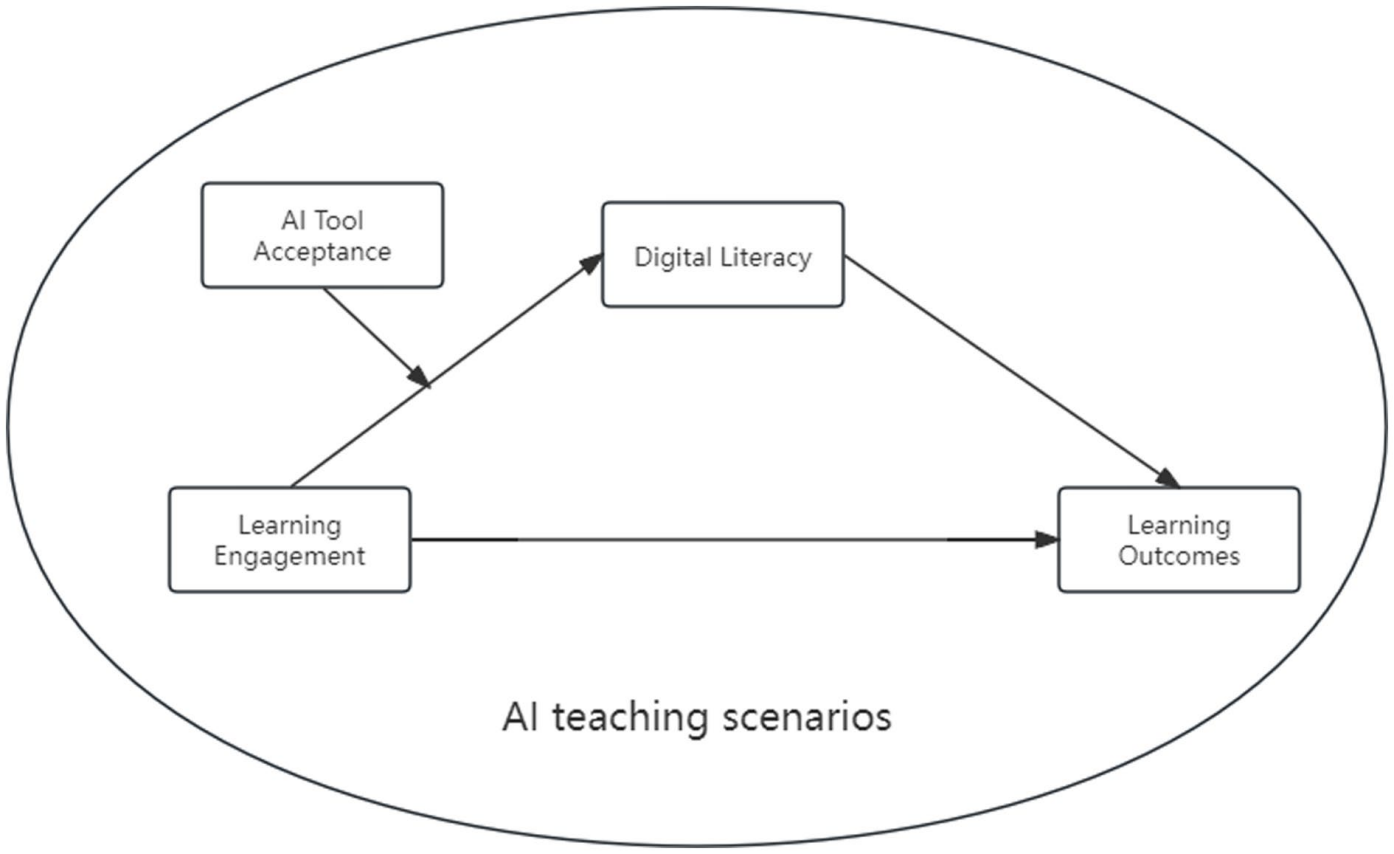
Research model.

The research framework of this paper is shown in Figure 1.

## 3 Research method

### 3.1 Data sources

This study employed a questionnaire survey method. The main research areas were Beijing, Tianjin, and Hebei Province, with a small number of students also surveyed from Henan Province and Shandong Province. Participants were full-time students majoring in public administration. The Beijing-Tianjin-Hebei region is China’s largest education-intensive area, while Shandong and Henan are also major provinces for the college entrance examination, with large student populations and numerous universities, making education highly dynamic.

This study conducted large-scale data collection using quantitative questionnaires (covering the Learning Engagement Scale, AI Tool Acceptance Scale, Digital Literacy Scale, and Learning Outcomes Scale) to initially verify the relationships among the variables. To reduce common method bias, questionnaire data were collected in two waves, with a half-month interval between them. The first wave measured demographic variables, independent variables, and mediating variables; the second wave measured moderating variables and the dependent variable. This approach helped avoid biases caused by habitual thinking when completing the questionnaires, further improving the authenticity and accuracy of the responses. In the first wave, 210 electronic questionnaires were distributed, and 200 valid questionnaires were returned, yielding an effective response rate of 95.24%. Half a month later, the team launched a second wave, distributing 215 questionnaires and eventually recovering 200 valid responses, with an effective response rate of 93.02%. Thus, a total of 400 valid questionnaires were collected in this study.

### 3.2 Measures

Regarding the four core variables—learning engagement, AI tool acceptance, digital literacy, and learning outcomes—this study considered the effectiveness of scale localization in China and ultimately selected scales from authoritative academic journals. Prior to use, translation and back-translation procedures were followed to ensure semantic equivalence between the Chinese and English versions, thereby guaranteeing the scientific rigor and adaptability of the measurement tools. Since the selected scales were originally in English, this study strictly adhered to the translation-back-translation procedure proposed by Brislin (1970) to ensure semantic, conceptual, and cultural equivalence between the Chinese version and the original. Specifically, three master’s students majoring in sociology independently translated the English scales into Chinese, producing three draft translations. Through comparative discussion of these drafts, an initial Chinese version was developed. Subsequently, three English major students independently back-translated the initial Chinese version into English. The back-translated version was then compared with the original version item by item to identify semantic discrepancies, and the Chinese expressions were revised accordingly to finalize the scales.

To accurately estimate the net effect of AI tools on the learning outcomes of public administration students, this study controlled for variables such as gender, age, grade level, and major direction in the model. Existing research indicates that, regarding gender, systematic differences between male and female students in the frequency of AI tool use, self-confidence, and ethical attitudes—if not controlled—may confound the AI intervention effect (Møgelvang et al., 2024). Age affects the maturity of learning strategies, self-regulation ability, and efficiency of knowledge absorption, and also reveals differences in preferences for AI tools and learning methods. Controlling for age can remove the interference of maturity differences on the AI effect. Grade level reflects students’ knowledge reserves, course difficulty, and ability progression during professional training. Students at different grade levels exhibit differences in adaptability and usage strategies for AI tools; controlling for grade level helps eliminate interference from cumulative learning effects and course variations. Major direction encompasses the differentiation among subfields within public administration. Students from different major directions show significant differences in course content, knowledge structures, and training goals, which in turn affect their preferences and abilities regarding AI tool acceptance and use. Controlling for major direction avoids confounding the AI effect due to disciplinary differences. In summary, including these four control variables helps to more precisely isolate the net effect of AI tools on learning outcomes.

The explanatory variable in this study is learning engagement. To measure this variable, the study adopted the Learning Engagement Scale proposed by Carmona-Halty et al. (2019). Representative items include: “As a student, I feel full of energy when using AI tools for learning” and “When I use AI tools to assist me in school or class, I feel energetic and capable.” A 5-point Likert scale was used, with higher scores indicating stronger learning engagement.

The mediating variable in this study is digital literacy. Consistent with the research topic, this study used the Digital Literacy Scale for college students developed by Acosta et al. (2024). This scale demonstrated good reliability and validity in the original study and employs a 5-point Likert scale. Representative items include: “I can use AI tools to obtain news and assist in learning about policy dynamics” and “I can accurately search for needed information on the Internet and complete learning tasks efficiently.”

To measure the moderating variable—AI tool acceptance—this study used the AI Acceptance Scale developed by Gansser and Reich (2021). Representative items include: “Using AI tools in public administration learning helps me complete tasks faster” and “For me, learning public administration with AI tools is easy.”

For the measurement of learning outcomes, this study utilized the operational scale proposed by Alavi et al. (2002), also employing a 5-point Likert scale, where higher scores indicate stronger learning outcomes. The authors made adaptive modifications to the scale for the purposes of this study and ultimately developed the final questionnaire.

## 4 Results

### 4.1 Descriptive statistics

Among the 400 valid questionnaires, 228 respondents were male, accounting for 57.0%, and 172 were female, accounting for 43.0%. Regarding age distribution, 39 respondents (9.8%) were under 18 years old; 235 (58.8%) were aged 18–25; 81 (20.3%) were aged 26–30; and 45 (11.3%) were over 30. In terms of grade level, 63 students (15.8%) were freshmen, 122 (30.5%) were sophomores, 64 (16.0%) were juniors, 53 (13.3%) were seniors, 36 (9.0%) were master’s students, and 62 (15.5%) were doctoral students. Regarding the distribution of respondents’ major directions, 141 (35.3%) were in administrative management, 182 (45.5%) were in public utilities management, and 77 (19.3%) were in other fields (Table 1).

**Table 1:** Demographic description.

Name	Options	Frequency	Percentage (%)
Sex	female	228	57.0
	male	172	43.0
Age	Under 18 years old	39	9.8
	18–25 years old	235	58.8
	26–30 years old	81	20.3
	Over 30 years old	45	11.3
Grade	Freshman	63	15.8
	Sophomore	122	30.5
	Junior	64	16.0
	Senior	53	13.3
	Master’s student	36	9.0
	Doctoral student	62	15.5
Major directions	Administrative management	141	35.3
	Public utilities management	182	45.5
	Others	77	19.3

### 4.2 Correlations

As shown in Table 2, in the context of AI embedded in teaching, learning engagement was significantly positively correlated with digital literacy and learning outcomes. Digital literacy also had a positive effect on learning outcomes. These findings support Hypotheses 1, 2, and 3. Specifically, in the AI-integrated teaching context, learning engagement was significantly and positively correlated with learning outcomes (*r* = 0.342, *p* < 0.05). Based on Cohen’s (2013) effect size criteria—where 0.10 ≤ |*r*| < 0.30 indicates a small effect, 0.30 ≤ |*r*| < 0.50 a medium effect, and |*r*| ≥ 0.50 a large effect (Cohen, 2013)—this correlation falls within the medium range, suggesting that learning engagement exerts a moderate positive influence on student learning outcomes. Similarly, in the same context, learning engagement demonstrated a medium-sized positive association with students’ digital literacy (*r* = 0.418, *p* < 0.05), while digital literacy exhibited a medium-sized positive association with learning outcomes (*r* = 0.374, *p* < 0.05).

**Table 2:** Correlations.

Variables	1	2	3	4	5	6	7	8
1. Sex	1							
2. Age	0.049	1						
3. Grade	0.040	0.893^**^	1					
4. Specialization	0.017	−0.030	−0.050	1				
5. Learning Engagement	0.069	−0.035	−0.065	−0.014	1			
6. Digital literacy	−0.035	0.036	0.007	−0.011	0.418^**^	1		
7. AI Tool Acceptance	0.0.014	0.052	0.033	−0.047	0.405^**^	0.420^**^	1	
8. Learning outcomes	−0.016	0.032	0.011	0.054	0.342^**^	0.374^**^	0.299^**^	1
Mean	1.58	2.33	3.16	1.84	3.32	3.39	3.30	3.45
SD	0.496	0.802	1.675	0.722	0.940	0.916	0.916	1.030

** *p* < 0.01.

### 4.3 Reliability test

Reliability analysis refers to the assessment of the consistency, stability, and reliability of the measured data. Internal consistency is commonly used to indicate the level of reliability. The reliability of the questionnaire was primarily evaluated by calculating Cronbach’s *α* coefficients for the scales. A larger Cronbach’s *α* coefficient indicates stronger internal consistency among the items within a scale, and thus higher reliability. When Cronbach’s *α* exceeds 0.7, it suggests good internal consistency. The results are presented in Table 3. The Cronbach’s α values for the four variables involved in this study were 0.933, 0.966, 0.955, and 0.868, respectively. All Cronbach’s *α* values were above 0.7, indicating that the scales and questionnaires used in this study had good internal consistency, and that the analytical results are highly reliable.

**Table 3:** Reliability analysis of various variables.

Variable	Cronbach’s alpha	Number of question items
Learning engagement	0.933	9
Digital literacy	0.966	18
AI tool acceptance	0.955	14
Learning outcomes	0.868	3

### 4.4 Confirmatory factor analysis

This study conducted confirmatory factor analysis (CFA) using Mplus 8.0. The actual data were fitted to the hypothesized model to test whether the theoretical model was consistent with the observed data. The analysis compared the baseline model (four-factor model) with a three-factor model, a two-factor model, and a one-factor model. The fit indices included the chi-square test statistic (*χ*^2^), RMSEA, CFI, and TLI, supplemented by significance tests of factor loadings to confirm the explanatory power and reliability of each variable corresponding to its factor. This procedure further enhanced the structural validity and predictive power of the model.

The results (shown in Table 4) indicated that the three-factor, two-factor, and one-factor models exhibited certain problems across the fit indices, suggesting a degree of mismatch between these models and the data. The baseline model was judged to be the most suitable for explaining the observed data, demonstrating good data structure validity.

**Table 4:** Confirmatory factor analysis.

Model	*χ* ^2^	df	*χ*^2^/df	CFI	TLI	RMSEA	SRMR
Baseline model (LE; DI; AAI; LO)	942.618	896	1.05	0.996	0.996	0.011	0.028
Three factor model (LE; DI + AAI; LO)	3,862.672	899	4.30	0.750	0.737	0.091	0.130
Two factor model (LE; DI + AAI + LO)	4,337.750	901	4.81	0.711	0.696	0.098	0.133
Single factor model (LE + DI + AAI + LO)	5,981.594	902	6.63	0.572	0.551	0.119	0.150

*N* = 400; LE, Learning Input; DI, Digital Literacy; AAI, Acceptance of AI Tools; LO, Learning Outcomes; CFI, Comparative Fit Index; TLI, Tucker–Lewis Index; RMSEA, Root Mean Square Error of Approximation; SRMR, Standardized Root Mean Square Residual.

The baseline model included four variables: learning engagement, digital literacy, AI tool acceptance, and learning outcomes. Its fit indices were as follows: *χ*^2^/df = 1.05 (<3), CFI = 0.996 (>0.9), TLI = 0.996 (>0.9), RMSEA = 0.011 (<0.08), and SRMR = 0.028 (<0.08). That is, all CFA indicators showed that the baseline model performed well on every fit index, indicating that it was the most suitable for explaining the observed data and possessed good data structure validity. The confirmatory factor analysis thus confirmed that the data fit the model well.

The three-factor model combined digital literacy and AI tool acceptance into one factor, while keeping learning engagement and learning outcomes as separate factors. Its fit indices were: *χ*^2^/df = 4.30 (>3), CFI = 0.750, TLI = 0.737 (both < 0.8), RMSEA = 0.091 (>0.08), and SRMR = 0.130 (>0.08). Hence, the three-factor model had a poorer fit compared to the baseline model.

The two-factor model combined digital literacy, AI tool acceptance, and learning outcomes into one factor, while keeping learning engagement as a separate factor. Its fit indices were: *χ*^2^/df = 4.81 (>3), CFI = 0.711, TLI = 0.696 (both < 0.8), RMSEA = 0.098 (>0.08), and SRMR = 0.133 (>0.08). Thus, the two-factor model also exhibited a poor fit relative to the baseline model.

The one-factor model combined all four variables into a single factor. Its fit indices were: *χ*^2^/df = 6.63, CFI = 0.572, TLI = 0.551, RMSEA = 0.119, and SRMR = 0.150. All indices indicated serious problems, meaning the one-factor model had a much poorer fit compared to the baseline model.

A comprehensive comparison revealed that the baseline model had the best fit, indicating that the current data possessed sound structural validity.

### 4.5 Common method Bias test

This article uses the Harman single factor analysis method to test the items of the four scales in this study on the collected data. The results showed that the variance explanation rate of the first factor was only 38.368%, which did not reach the critical value (40%). That is, the impact of common method bias on the research data was within a reasonable range, and there was no serious problem of common method bias (Table 5).

**Table 5:** Common method deviation.

Ingredient	Initial eigenvalue			Extract the sum of squared loads					
	Total	Variance percentage	Accumulated%	Total	Variance percentage	Accumulated%	Variables	Total	Variance percentage
1	16.882	38.368	38.368	16.882	38.368	38.368	11.313	25.710	25.710
2	5.767	13.107	51.475	5.767	13.107	51.475	8.911	20.253	45.964
3	3.980	9.045	60.519	3.980	9.045	60.519	5.977	13.584	59.547
4	1.879	4.271	64.790	1.879	4.271	64.790	2.307	5.243	64.790
…	…	…	…	…	…	…	…	…	…
44	0.176	0.400	100.000						

### 4.6 Analysis of the mediating effect of digital literacy between learning engagement and learning outcomes

This study hypothesized that in the context of AI embedded in teaching, digital literacy mediates the relationship between learning engagement and learning outcomes among public administration majors (Hypothesis 4). Mediating effects can be classified into two types: complete mediation and partial mediation. Complete mediation occurs when the effect of the predictor variable on the dependent variable is transmitted entirely through the mediating variable. In this case, after including the mediating variable, the direct effect of the predictor on the dependent variable becomes non-significant, and the predictor’s influence is fully dependent on the mediating variable. That is, when the direct effect is non-significant but the indirect effect is significant, a complete mediation effect is established. Partial mediation occurs when the effect of the predictor on the dependent variable is partially transmitted through the mediating variable. In this case, after including the mediating variable, the direct effect remains significant, meaning the predictor affects the dependent variable both directly and indirectly. That is, when both the direct and indirect effects are significant, a partial mediation effect is established.

To test the mediating role of digital literacy between learning engagement and learning outcomes, this study examined the relationships between learning engagement and learning outcomes, between learning engagement and digital literacy, and between digital literacy and learning outcomes.

The results (Table 6) showed that for the path “learning engagement → learning outcomes” (denoted as a), the direct effect coefficient was 0.387 (*p* < 0.01), with a standard error of 0.050, and the 97.5% BootCI confidence interval was (0.288, 0.485). For the path “learning engagement → digital literacy” (denoted as b), the direct effect coefficient was 0.609 (*p* < 0.01), with a standard error of 0.037, and the 97.5% BootCI confidence interval was (0.534, 0.680). For the path “digital literacy → learning outcomes” (denoted as c), the direct effect coefficient was 0.385 (*p* < 0.01), with a standard error of 0.049, and the 97.5% BootCI confidence interval was (0.291, 0.481).

**Table 6:** Mediating effect test.

Item	Estimate	S.E.	*p*	97.5% BootCI	
Learning engagement → learning outcomes	0.387	0.050	0.000	0.288	0.485
Learning engagement → digital literacy	0.609	0.037	0.000	0.534	0.680
Digital literacy → learning outcomes	0.385	0.049	0.000	0.291	0.481
Learning engagement → digital literacy → learning outcomes	0.235 (Indirect effect)	0.033	0.000	0.171	0.302
Learning engagement → digital literacy → learning outcomes	0.622 (Total effect)	0.038	0.000	0.547	0.695

For the mediating path “learning engagement → digital literacy → learning outcomes” (a*b), the indirect effect value was 0.235 (*p* < 0.01), with a standard error of 0.033, and the 97.5% BootCI confidence interval was (0.171, 0.302), which did not include 0, indicating that the indirect effect was significant and of moderate magnitude. The total effect was 0.622 (*p* < 0.01), with a standard deviation of 0.038, and the 97.5% BootCI confidence interval was (0.547, 0.695), which did not include 0, indicating that the total effect was significant.

In conclusion, both the direct and indirect effects were significant. Thus, the mediating path was supported and constituted a partial mediation. Hypothesis 4 is therefore verified; that is, digital literacy plays a mediating role in the relationship between learning engagement and learning outcomes. Specifically, in the context of AI embedded in teaching, the learning engagement of students majoring in public administration leads to an improvement in their learning outcomes by enhancing their digital literacy.

### 4.7 Analysis of the moderating effect of AI tool acceptance on the relationship between learning engagement and digital literacy

The Bootstrap method implemented in PROCESS was used to analyze the moderating effect of AI tool acceptance. The number of bootstrap iterations was set at 5,000, and the confidence interval was 95%. As shown in Table 7, the interaction effect between learning engagement and AI tool acceptance was significantly positive, indicating that AI tool acceptance positively moderates the effect of learning engagement on digital literacy (*β* = 0.204, *p* < 0.01). This moderating effect is of moderate magnitude. Specifically, in the context of AI embedded in teaching, among public administration students with higher levels of AI tool acceptance, the positive predictive effect of learning engagement on digital literacy is stronger. Therefore, Hypothesis 5 was supported.

**Table 7:** Analysis of the moderating effect of AI tool acceptance.

Item	Learning engagement (LE)						Digital literacy (DI)					
	*β*	S.E.	*t*	*p*	LLCI	ULCI	*β*	S.E.	*t*	*p*	LLCI	ULCI
Sex	0.071	1.411	0.159	−0.053	0.321	0.071	−0.103	0.079	−1.301	0.194	−0.258	0.523
Age	0.112	1.005	0.315	−0.125	0.387	0.112	0.123	0.108	1.147	0.252	−0.089	0.337
Grade	−0.169	−1.518	0.130	−0.217	0.028	−0.169	−0.045	0.052	−0.861	0.390	−0.147	0.057
Specialization	−0.020	−0.396	0.692	−0.154	0.102	−0.020	−0.008	0.054	−0.138	0.891	−0.114	0.099
Learning engagement (LE)							0.252	0.047	5.403	0.000	0.160	0.343
AI tool acceptance (AAI)							0.259	0.048	5.446	0.000	0.165	0.352
LE × AAI							0.204	0.046	4.445	0.000	0.114	0.294
	*R* = 0.111, *R*^2^ = 0.012						*R* = 0.539, *R*^2^ = 0.291					
	*F* = 1.226, *p* = 0.299						*F* = 22.971, *p* = 0.000					

**Figure 2:**
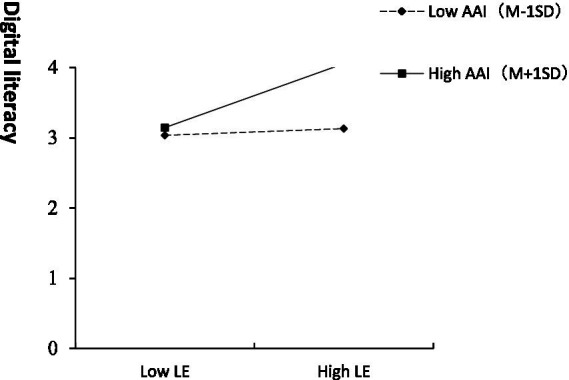
The moderating role of AI tool acceptance on the relationship between learning engagement and digital literacy.

To further verify the moderating effect of AI tool acceptance, this study also employed simple slope analysis to examine the effect of learning engagement on digital literacy at different levels of AI tool acceptance. At a low level of AI tool acceptance (one standard deviation below the mean), the effect of learning engagement on digital literacy was not significant, as the 97.5% bootstrap confidence interval (BootCI) contained zero. At a high level of AI tool acceptance (one standard deviation above the mean), the positive effect of learning engagement on digital literacy became significantly stronger (*β* = 0.438). As shown in Figure 2, the higher the level of AI tool acceptance, the stronger the positive relationship between learning engagement and digital literacy. Thus, Hypothesis 5 was further supported.

## 5 Conclusion and discussion

### 5.1 Discussion of results

This study identified a significant positive correlation between learning engagement and learning outcomes. This finding aligns with the conclusion drawn by Carini et al. (2006) in the context of traditional higher education and extends the work of Saqr et al. (2023) on the longitudinal dynamic association between learning engagement and academic achievement. It is noteworthy, however, that the effect size in this study slightly differs from the data reported by Wong et al. (2024) in related research. This discrepancy may be attributed to the highly context-dependent and policy-sensitive nature of public administration course content. When using AI tools for learning, students in this field must allocate additional cognitive resources for critical judgment, which may influence the impact of engagement on outcomes. The mediating role of digital literacy (indirect effect = 0.235) is a core finding of this study. This result supports the view of Phon and Ali (2025) that “digital literacy significantly affects student engagement and perception of quality education.” Compared to the findings of Su et al. (2023) in an online learning context, the effect proportion here is higher. A potential explanation is that public administration learning relies heavily on information acquisition, policy text analysis, and data interpretation—tasks that represent core application scenarios for digital literacy. Based on these findings, the following recommendations are proposed:

First, embed AI technology into core courses. It is advisable to design modules such as “AI-assisted policy simulation” and “AI tool-aided official document writing” within core public administration curricula. This approach directly links AI tool usage to professional tasks, thereby enhancing the quality of learning engagement.

Second, implement stratified cultivation of digital literacy. Training for lower-grade students should focus on foundational digital literacy, such as information retrieval and evaluation. For higher-grade students, the focus should shift to advanced competencies, including AI ethics judgment and algorithm critique.

Third, adopt training strategies that enhance AI tool acceptance. Prior to promoting AI tools, initiatives such as case discussions and sharing success stories can help reduce students’ technical anxiety and increase their perception of the tools’ usefulness.

### 5.2 Limitations and future research directions

This study has several limitations that point to directions for future research.

First, self-report bias. Although this study utilized two separate questionnaires and Harman’s single-factor test, all variables were derived from student self-reports. Self-assessed learning outcomes may deviate from objective academic performance. This is particularly likely among students with high AI tool acceptance, who may overestimate the learning improvement attributed to AI tools. Future research could incorporate teacher evaluations, course grades, or learning analytics data as more objective criteria.

Second, cultural and regional sample limitations. The sample was primarily drawn from the Beijing-Tianjin-Hebei region, with a smaller portion from Henan and Shandong. These areas have dense higher education resources and relatively complete digital infrastructure, providing students with more exposure to AI tools compared to those in central and western regions. Consequently, the conclusions may not be directly generalizable. Future studies should employ nationwide stratified sampling and examine whether regional digital development levels serve as a moderating variable.

Third, the cross-sectional design cannot establish strict causality. Although a mediated moderation model was constructed based on theoretical hypotheses, statistical paths are not equivalent to temporal causal relationships. The longitudinal study by Saqr et al. (2023) confirmed a dynamic interaction between learning outcomes and learning engagement. This study cannot rule out reverse causality. Future work should adopt a longitudinal design with three or more waves (e.g., measuring once per semester over two academic years) and use cross-lagged panel models or latent growth models to examine the temporal ordering of variables.

Fourth, limitations of the measurement scales. The learning engagement scale (Carmona-Halty et al., 2019) and the digital literacy scale (Acosta et al., 2024) used herein demonstrate good reliability and validity. However, their items are designed for general digital learning environments and are not specifically tailored to AI tool usage scenarios. Furthermore, the measurement of AI tool acceptance employed a general scale that does not distinctly capture dimensions such as perceived usefulness, perceived ease of use, and social influence. Future research should develop an AI literacy scale specific to the public administration context and examine the moderating effects of its sub-dimensions. Additionally, the measurement of “learning outcomes” in this study was relatively limited (Alavi et al., 2002), focusing mainly on perceived learning gains and lacking coverage of practical skills and social responsibility, which are highly valued in public administration. Future studies are advised to incorporate objective performance assessments.

## Data Availability

The original contributions presented in the study are included in the article/supplementary material, further inquiries can be directed to the corresponding author.
